# Supplementary material for: A high-resolution mRNA expression time course of embryonic development in zebrafish
Source: eLife. 2017 Nov 16;6:e30860. doi: 10.7554/eLife.30860 (PMC5690287; doi:10.7554/eLife.30860)
Supplement: Supplementary file 6. [file elife-30860-supp6.zip › biolayout-clusters-files/Cluster209-genes.html]

Cluster209


# Cluster209: Genes

| Ensembl ID | Gene Name | Chr | Start | End | Biotype |
| --- | --- | --- | --- | --- | --- |
| ENSDARG00000012827 | mettl3 | 7 | 21610210 | 21620772 | protein\_coding |
| ENSDARG00000069326 | mus81 | 7 | 19598946 | 19628298 | protein\_coding |
| ENSDARG00000071694 | ndc80 | 12 | 10315490 | 10328126 | protein\_coding |
| ENSDARG00000090820 | slc2a10 | 11 | 2344819 | 2355462 | protein\_coding |
| ENSDARG00000040538 | spsb4b | 15 | 41713346 | 41732585 | protein\_coding |
| ENSDARG00000103977 | terf2ip | 22 | 34623898 | 34638196 | protein\_coding |
| ENSDARG00000007315 | tmpoa | 4 | 16868746 | 16887360 | protein\_coding |
